# Brain-derived neurotrophic factor serum levels correlate with cognitive performance in Parkinson’s disease patients with mild cognitive impairment

**DOI:** 10.3389/fnbeh.2015.00253

**Published:** 2015-09-15

**Authors:** Alberto Costa, Antonella Peppe, Giovanni Augusto Carlesimo, Silvia Zabberoni, Francesco Scalici, Carlo Caltagirone, Francesco Angelucci

**Affiliations:** ^1^Niccolò Cusano UniversityRome, Italy; ^2^IRCCS, Fondazione Santa LuciaRome, Italy; ^3^Tor Vergata UniversityRome, Italy

**Keywords:** BDNF, Parkinson’s disease, cognitive functions, mild cognitive impairment, neuropsychological deficits

## Abstract

Brain-derived neurotrophic factor (BDNF) is a trophic factor regulating cell survival and synaptic plasticity. Recent findings indicate that BDNF could be a potential regulatory factor for cognitive functioning in normal and/or neuropathological conditions. With regard to neurological disorders, recent data suggest that individuals with Parkinson’s disease (PD) may be affected by cognitive deficits and that they have altered BDNF production. Therefore, the hypothesis can be advanced that BDNF levels are associated with the cognitive state of these patients. With this in mind, the present study was aimed at exploring the relationship between BDNF serum levels and cognitive functioning in PD patients with mild cognitive impairment (MCI). Thirteen PD patients with MCI were included in the study. They were administered an extensive neuropsychological test battery that investigated executive, episodic memory, attention, visual-spatial and language domains. A single score was obtained for each cognitive domain by averaging *z*-scores on tests belonging to that specific domain. BDNF serum levels were measured by enzyme-linked immunoassay (ELISA). Pearson’s correlation analyses were performed between BDNF serum levels and cognitive performance. Results showed a significant positive correlation between BDNF serum levels and both attention (*p* < 0.05) and executive (*p* < 0.05) domains. Moreover, in the executive domain we found a significant correlation between BDNF levels and scores on tests assessing working memory and self-monitoring/inhibition. These preliminary data suggest that BDNF serum levels are associated with cognitive state in PD patients with MCI. Given the role of BDNF in regulating synaptic plasticity, the present findings give further support to the hypothesis that this trophic factor may be a potential biomarker for evaluating cognitive changes in PD and other neurological syndromes associated with cognitive decline.

## Introduction

Brain-derived neurotrophic factor (BDNF) belongs to the family of neurotrophins that regulate the survival and functioning of neurons in the central nervous system (Baydyuk and Xu, 2014). BDNF was originally described as a trophic factor for dopaminergic neurons of the substantia nigra (Hyman et al., 1991). However, successive studies found that its action on neurons is not only trophic but involves the regulation of synaptic plasticity through the activation of signal transduction pathways that influence the release of neurotransmitters such as glutamate and GABA (Gottmann et al., 2009). These effects on synaptic transmission have been associated with the long-term potentiation that takes place in the hippocampus during learning and memory processes (Leal et al., 2014). Thus, BDNF not only has a trophic action but also the potential to regulate cognitive processes.

In recent years, the latter role of BDNF was confirmed in both animal and human studies. In rodent models, it was shown that knockout of the BDNF gene affects spatial learning and memory (Gorski et al., 2003; Monteggia et al., 2004; Heldt et al., 2007), whereas transgenic overexpression of BDNF in cortical and subcortical (i.e., hippocampus) regions sustains performance (Koponen et al., 2004). Li et al. (2012) also documented that, in cognitively affected rats, BDNF infusion into the nucleus accumbens improved cognition, synaptic plasticity and cell signaling. As for human data, in both healthy individuals (Alfimova et al., 2012) and in persons with psychiatric disturbances (Lu et al., 2012; Tükel et al., 2012), BDNF gene polymorphism (Val66Met) was found to be associated with executive dysfunctions. In a sample of more than 50 healthy young subjects, Koven and Collins (2014) recently documented a significant correlation between (urinary) BDNF concentration and cognitive performance on tests tapping cognitive flexibility (i.e., Trail Making Test, Design Fluency and Verbal Category Switching). Nonetheless the role of BDNF gene polymorphism in Parkinson’s disease (PD) is not well defined and the results are sometimes contradictory. While some authors report an association between gene polymorphism with cognitive impairments in PD patients (Foltynie et al., 2005; Guerini et al., 2009) or with the onset of the disease (Karamohamed et al., 2005), other studies do not show such correlations (Dai et al., 2013; Svetel et al., 2013; Białecka et al., 2014). Many of these studies however are not strictly comparable in term of disease stages and the methodology used to assess cognitive impairments (Alonso-Navarro et al., 2014; Białecka et al., 2014). Thus, the results do not permit definitive conclusions. Larger sample-size and multiethnicity studies with homogeneous PD patients and well-matched controls are needed in the future study.

Above data indicate that BDNF could be a potential regulatory factor for cognitive functioning in normal and/or neuropathological conditions. Among the neurological disorders affected by cognitive deficits, PD is interesting because the neuronal pathways affected are involved in some cognitive functions that respond to the action of BDNF (Murer et al., 2001; Guillin et al., 2003). In fact, PD is characterized by dopamine cell loss within the substantia nigra that causes a precocious deafferentation of the nigro-striatal dopamine pathway with later involvement of the meso-cortical and mesolimbic dopamine pathways (Yeterian and Pandya, 1991; Jahanshahi et al., 2010). Thus, in addition to motor disorders PD patients may also present cognitive deficits including mild cognitive impairment (MCI) and dementia (Robbins and Cools, 2014). Typically PD patients with cognitive deficits have a dysexecutive profile that is characterized by poor performance on tests tapping shifting and planning (Cools, 2006; Cools and D’Esposito, 2011; MacDonald and Monchi, 2011), working memory (Cools, 2006; Cools and D’Esposito, 2011) and free recall of studied information (Costa et al., 2014a). As stated before, BDNF is a trophic factor for the dopaminergic neurons of these brain regions. Indeed, many studies have shown that PD patients show decreased BDNF levels in the substantia nigra (pars compacta; Howells et al., 2000) and in serum (Scalzo et al., 2010). Moreover, BDNF serum concentration was associated to the level of dopamine transporter binding in the striatum (Ziebell et al., 2012).

Studies on cerebrospinal fluid (CSF) of PD patients revealed an association between BDNF and cognitive performance (Leverenz et al., 2011) and increased BDNF levels compared with controls (Salehi and Mashayekhi, 2009). Moreover, CSF BDNF levels were found to be reduced in PD patients with major depression (MD) as compared to solely depressed patients after treatment with antidepressants (citalopram; Pålhagen et al., 2010). Despite these studies, the role of CSF BDNF levels as biomarker in PD needs to be replicated according to more strictly diagnostic standardized criteria and in longer follow- up periods (Jiménez-Jiménez et al., 2014).

Altogether these findings suggest that BDNF serum levels may not only be a trait that characterizes dopaminergic dysfunctions in PD patients but could also be a potential biomarker of their cognitive deficits. To test this hypothesis, we performed a pilot study with a groups of individuals with PD and MCI, in which we assessed BDNF serum levels and correlated them with scores obtained on tests measuring episodic memory, language, attention, executive and visual-spatial functions. Since in humans BDNF has been reported to correlate with specific components of the executive domain that are precociously weakened in PD (Koven and Collins, 2014), we expected to find a significant association between BDNF serum levels and PD patients’ performance on executive tests.

## Materials and Methods

### Patients

We recruited 13 right-handed subjects with idiopathic PD who gave their written informed consent. The study was approved by Ethics Committee of the Santa Lucia Foundation. The United Kingdom PD Society brain bank criteria were used to diagnosis PD (Hughes et al., 1992).

MCI was diagnosed according to the criteria of Litvan et al. (2012). Accordingly, to be included PD subjects patients have a performance that was 1.5 SD below the average performance from the average performance of the normal population on at least one neuropsychological test investigating executive abilities and on another test sensitive to short-term memory/attention, visual-spatial abilities, episodic memory and language (the neuropsychological test battery is described below). The Mini-Mental State Examination (MMSE; Folstein et al., 1975) score had to be ≥26. In order to exclude psychiatric diseases, neurological conditions other than PD, vascular brain damage and major systemic or metabolic pathologies that could influence cognitive performance, we performed neuropsychiatric, neuroradiological (CT or MR) and laboratory examinations.

In order to assess functional abilities in daily living and their relationship with cognitive functioning we administered the Clinical Dementia Rating Scale, the Instrumental Activities of Daily Living scale (Lawton and Brody, 1969) and the Pill questionnaire (Dubois et al., 2007). Severity of depressive and apathy symptoms was also assessed by administering the Beck Depression Inventory (Beck et al., 1961; Visser et al., 2009) and the Apathy Evaluation Scale–self version (Marin et al., 1991; Leentjens et al., 2008), respectively. PD patients were being treated with levodopa and/or dopamine agonists (ropinirole, pramipexole and rotigotine) during examination period. The levodopa equivalent and the clinical and sociodemographic characteristics of the group are reported in Table 1. Twenty healthy controls (HC) were also recruited. Inclusion criteria for HC were: (i) absence of subjective cognitive deficits and (ii) MMSE score ≥26 (Folstein et al., 1975). Exclusion criteria were: (i) performance 1.5 SD below the normative population on any test of the standardized neuropsychological screening battery; (ii) current or previous neurological or psychiatric disorders, major systemic or metabolic diseases that could potentially affect cognitive functioning; and (iii) taking medications that have an effect on brain functioning. HC were only administered the behavioral tests.

**Table 1:** **Socio-demographic and clinical characteristics of the samples**.

	Mean (SD)	
	PD patients	Healthy controls	*F* values	*p* values
Age	68.3 (7.8)	66.4 (7.4)	0.48	>0.40
Years of formal education	11.2 (4.9)	12.4 (3.3)	0.71	>0.40
Mini mental state examination	28.1 (1.5)	29.4 (0.7)	12.1	0.001
Pill questionnaire	3.3 (0.75)
ADL	5.2 (1.3)
IADL	7.1 (1.5)
Beck depression inventory	7.8 (5.6)	6.7 (5.8)	0.25	>0.60
Apathy evaluation scale	32.3 (6.8)	28.0 (6.1)	3.05	0.093
Disease duration	8.8 (6.9)
Daily levodopa equivalents	695 (294)
UPDRS part-III	26.5 (11.1)	

*UPDRS, Unified Parkinson’s Disease Rating Scale*.

Socio-demographic and clinical characteristics of individuals participating to the study are reported in Table 1.

### Neuropsychological Test Battery

The following neuropsychological tests were administered to subjects according to the domain they assess: (a) Episodic memory: Immediate and Delayed Recall of a 15-Word List (Carlesimo et al., 1996); Prose Recall (Carlesimo et al., 2002); Immediate and delayed reproduction of Rey’s Figure (Carlesimo et al., 2002); (b) Attention and short-term memory: Digit Span and Corsi Block Tapping test Forward and Backward (Monaco et al., 2013); Trail Making Test -Part A (Giovagnoli et al., 1996); (c) Executive functions: Phonological Word Fluency (Carlesimo et al., 1996); Modified Card Sorting test (MCST; Nocentini et al., 2002); Raven’s Coloured Progressive Matrices (Carlesimo et al., 1996); Trail Making Test–Part B (Giovagnoli et al., 1996); (d) Language: Objects and Verbs Naming subtests from the Neuropsychological Examination of Aphasia (Capasso and Miceli, 2001); and (e) Visual-spatial functions: Copy of Drawings and Copy of Drawings with Landmarks (Carlesimo et al., 1996); Copy of Rey’s Figure (Carlesimo et al., 2002).

Additional tests were administered to assess executive functioning according to the ability they investigated: Zoo Map test (Wilson et al., 1998; planning and self-monitoring), Stroop test (Barbarotto et al., 1998; response inhibition and self-monitoring), Alternate Phonemic and Category fluency (Cognitive Flexibility/shifting) and Category Fluency (Costa et al., 2014c).

In order to standardize the measurement scales between the different tests, raw scores were transformed into *z*-scores by considering the means and standard deviations of the HC group.

First, a unique *z*-score was computed for each cognitive domain (i.e., episodic memory, attention and short-term memory, executive functions, language and visual-spatial domains) by averaging subjects’ *z*-scores on tests belonging to that specific domain.

Following Koven and Collins (2014), to investigate the possible correlation between BDNF levels and specific components of the executive domain (abstract reasoning/planning, self-monitoring/response inhibition, cognitive flexibility, fluency and working memory), individual *z*-scores were computed for each executive component by averaging the subjects’ *z*-scores on the relative tests. Thus, an individual score was obtained for abstract reasoning/planning (total accuracy score on the Zoo Map Test; number of categories achieved on the MCST; accuracy score on the Progressive Matrices), Self-monitoring/Response inhibition (Errors made on the Zoo Map Test; errors made and response times on interference condition of the Stroop test), Cognitive Flexibility (perseverative errors committed on the MCST; accuracy score on Alternate Phonemic and Semantic fluency; Trail Making Test Part B-Part A score), Fluency (Phonemic and Semantic fluency), and Working memory (Digit and Corsi span backward). All *z*-scores were considered as absolute values so that higher scores corresponded to better performance.

### Blood Sampling

Blood samples were collected between 8:00 a.m. and 10:00 a.m., centrifuged at 2000×g for 20 min and stored at −80°C until analysis (Angelucci et al., 2015).

#### Determination of BDNF Content

BDNF was detected in sandwich enzyme-linked immunoassay (ELISA; R&D Systems, USA; cat. N° DY248) according to the manufacturer’s instructions as previously described (Angelucci et al., 2015). Wells were developed with tetramethylbenzidine and measured at 450/570 nm and BDNF content was quantified against a standard curve calibrated with known amounts of protein. The detection limit for BDNF was 15 pg/ml. Measurements were performed in duplicate and values are expressed as ng/ml. Cross-reactivity with other related trophic factors (NGF, NT-3; NT-4; TGFβ, TGFα) was less than 3% (Angelucci et al., 2015).

### Statistical Analysis

In order to compare PD patients’ and HCs’ scores on the five main cognitive domains (episodic memory, attention and short-term memory, executive functions, language, visual-spatial functions), a multivariate analysis of variance (MANOVA) and individual univariate ANOVAS were carried out.

Pearson’s r correlations were performed to examine the relationship between BDNF serum levels and *z*-scores obtained by PD patients in the different cognitive domains examined.

The same statistical analysis was used to examine the correlation between BDNF serum levels and PD patients’ *z*-scores on the different executive components.

## Results

### Comparisons Between PD Patients and Healthy Controls

**Figure 1:**
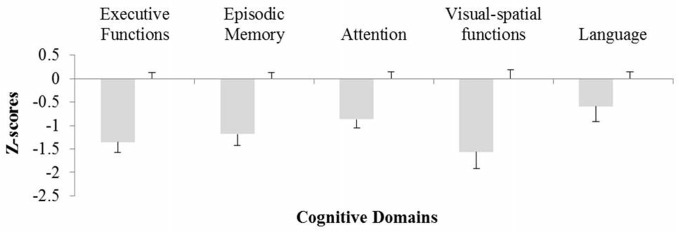
**Parkinson’s disease (PD) patients’ *z*-scores on the different cognitive domains examined**. The *x* axis represents healthy controls (HC) values (averaged). Vertical bars represent standard errors.

The *z*-scores obtained by the experimental subjects are illustrated in Figure 1. The MANOVA showed a significant effect (*F*_(5,27)_ = 9.64; *p* < 0.001). Subsequent univariate analyses showed that PD patients obtained significantly lower *z*-scores than HC in the cognitive domains of memory, executive functions, attention and visual-spatial abilities (*F*_(1,33)_ range: 17.1–32.5; *p* < 0.001 in all cases). By contrast, in the language domain the between group difference only approached statistical significance (*F*_(1,33)_ = 3.44; *p* = 0.073).

### Correlation Between BDNF Serum Levels and PD Patients’ Cognitive Scores

#### Analysis Involving the Five Main Cognitive Domains

Results of Pearson’s *r* correlation analyses showed a significant positive correlation between BDNF serum levels and PD patients’ *z*-scores in the executive (*r* = 0.62; *p* = 0.023) and attention (*r* = 0.59; *p* = 0.032) domains. This indicates that better scores on the above tests were associated with a higher concentration of BDNF. The effects related to analyses involving the remaining three cognitive domains did not reach statistical significance (episodic memory: *r* = −0.27; *p* = 0.36; visual-spatial domain: *r* = 0.33; *p* = 0.26; language domain: *r* = −0.05; *p* = 0.88).

### Analyses Involving the Different Components of the Executive Domain

A significant positive correlation was found between BDNF levels and *z*-scores referring to self-monitoring/response inhibition (*r* = 0.68; *p* = 0.011) and working memory (*r* = 0.62; *p* = 0.025) performance, indicating that higher BDNF levels were associated with better cognitive performance. The remaining analyses showed no significant effects (Planning/Abstraction: *r* = 0.48; *p* = 0.098; shifting: *r* = 0.41; *p* = 0.17; Fluency: *r* = 0.06; *p* = 0.85).

## Discussion

This study investigated the association between cognitive functioning and BDNF serum level in PD patients with MCI. For this purpose, we administered an extensive neuropsychological battery to a sample of PD patients including tests for the assessment of episodic memory, executive functions, attention, language and visual-spatial abilities. The results showed a positive association between BDNF serum levels and performance on neuropsychological tests investigating executive functioning and attention. Moreover, other analyses focusing on the different components of the executive domain showed a closer association between BDNF levels and working memory and self-monitoring/inhibition.

Our results are congruent with previous data reported in both animal and human studies that documented an association between BDNF-related activity and cognitive functioning (Koven and Collins, 2014; Robbins and Cools, 2014), particularly with data suggesting a specific association with abilities in the executive system domain (Gajewski et al., 2011; Alfimova et al., 2012; Lu et al., 2012; Tükel et al., 2012; Koven and Collins, 2014). In fact, molecular studies reported that the BDNF gene polymorphism (Val66Met) may affect executive functions in healthy subjects (Alfimova et al., 2012) and in patients with psychiatric disturbances (Lu et al., 2012; Tükel et al., 2012). Very interestingly, Koven and Collins (2014), who adopted a design similar to ours, recently documented a significant relationship in healthy subjects between (urinary) BDNF concentration and performance on executive tests. These authors examined a sample of more than 50 healthy young subjects who were administered a neuropsychological test battery investigating different subcomponents of the executive system. Adopting a correlational design, they demonstrated that subjects’ performance on tests tapping cognitive flexibility (i.e., Trail Making Test, Design Fluency and Verbal Category Switching) was positively associated with BDNF urinary concentrations. Our results differed from those of Koven and Collins in that we did not find a specific association between BDNF and set-shifting (although the relative correlation was in the expected direction). In fact, BDNF serum concentration correlated with the executive scores of working memory, self-monitoring and abstraction/planning. The imperfect congruence between our results and those of Koven and Collins’ study is likely due to differences in the recruited samples. Indeed, the participants in Koven and Collins’ study were healthy young individuals. Instead, our sample consisted of PD patients with a known cognitive weakness (i.e., MCI with a predominantly dysexecutive and amnestic neuropsychological profile).

BDNF plays a role in the promotion of the survival and function of striatal dopaminergic neurons and in regulating synaptic connectivity (Gómez-Palacio-Schjetnan and Escobar, 2013; He et al., 2013). Other studies have shown that BDNF brain and peripheral levels are decreased in PD patients as compared to HC (Scalzo et al., 2010). Moreover, Gyárfás et al. (2010) demonstrated that treatment with antiparkinsonian drugs may rise BDNF levels. As previously mentioned, PD is characterized by altered functioning of the brain dopamine systems, which involve the nigro-striatal, meso-cortical and mesolimbic pathways (Robbins and Cools, 2014). More specifically, dopamine deafferentation primarily involves the nigro-dorsal striatum pathways and dopamine projections to the dorsal prefrontal cortex (Baydyuk and Xu, 2014). In this regard, it has been hypothesised that in the early stages of PD dopamine altered activity is associated with cognitive weakness due to the regional distribution of dopamine receptor dysfunctioning (Cools and D’Esposito, 2011). In fact, dopamine depletion has an early effect on the striatal regions that are rich with D2 receptors, whose phasic activity is important for sustaining cognitive flexibility processes (Camps et al., 1990; Yeterian and Pandya, 1991; Agid et al., 1993; MacDonald and Monchi, 2011). Only later it involves the dorsal prefrontal cortex where tonic D1 activity sustains the ability to retain stable representations in the face of incoming information (i.e., resistance to interference, self-monitoring; Cohen et al., 2002; Frank, 2005; Costa et al., 2009, 2014b; Cools and D’Esposito, 2011). In fact, weakness of the executive system is a frequent and precocious finding in PD (Dirnberger and Jahanshahi, 2013). Therefore, our finding of a significant correlation between BDNF serum levels and prefrontal-related cognitive performance, although preliminarily, supports the hypothesis that BDNF activity contributes to maintaining normal prefrontal cortex functioning (Savitz et al., 2006; Woo and Lu, 2006; Galloway et al., 2008). In addition, the data presented here, together with the observation that BDNF is reduced in the brain and in the serum of PD patients, could suggest that the measurement of BDNF in serum may be used as a biological correlate of cognitive as well as motor functioning. The fact that BDNF levels positively correlate with performance on neuropsychological tests is encouraging. Although we demonstrated in a previous work that a cognitive rehabilitation protocol was able to increase BDNF serum levels and cognitive functions in PD patients affected by MCI, we did not find a significant correlation between the biological and neuropsychological data (Angelucci et al., 2015). As the number of patients was even smaller (7/8 patients per group) and only one measure of executive functioning was used, we can speculate that the lack of correlation in that study could have been due to both insufficient sample size and low sensitivity of the cognitive measure used to measure executive dysfunction in these patients.

Nonetheless, some limitations should be taken into account in the interpretation of our data. The study is limited by the poor sample size. Furthermore, we are merely describing an association between BDNF serum and cognitive performance and not a causal relationship. PD is now considered a multi-systemic neurodegenerative disorder that is characterized by a combination of motor and non-motor symptoms (Stacy, 2011). For a long time the main clinical focus in PD has been on the motor symptoms resulting from degeneration of the substantia nigra and the dopaminergic nigrostriatal pathway, but there is increasing recognition that other brain areas are involved in the manifestation of non motor symptoms (Rana et al., 2015) including the cerebral cortex (Lindenbach and Bishop, 2013), brainstem (Greene, 2014) and peripheral nervous system (Conte et al., 2013). Thus, alterations of BDNF brain and peripheral levels may occur in response to other deficits in these PD associated brain areas. This may limit the possibility to use BDNF as a possible marker of PD cognitive disturbances. Future studies, such as those using PET (Positron Emission Tomography), are needed to establish a direct correlation between dopaminergic denervation and BDNF.

In conclusion, this study shows that BDNF serum levels correlate positively with performance on neuropsychological tests investigating executive functioning and attention. These findings support the idea that this protein may represent a possible peripheral marker of cognitive functioning, although this hypothesis needs to be confirmed in larger cohorts of samples and with different methodologies.

## Author Contributions

AC, AP made substantial contributions to the conception and design of the work, and the acquisition, analysis, and interpretation of data for the work; GAC made substantial contributions to the conception and design of the work; SZ and FS made substantial contributions to the the acquisition of data for the work; CC made substantial contributions to the conception and design of the work; FA made substantial contributions to the conception of the work, and to the acquisition, analysis, and interpretation of data for the work; all drafted the work and revised it critically for important intellectual content; gave final approval of the version to be published; and agreed to be accountable for all aspects of the work in ensuring that questions related to the accuracy or integrity of any part of the work were appropriately investigated and resolved.

## Conflict of Interest Statement

The authors declare that the research was conducted in the absence of any commercial or financial relationships that could be construed as a potential conflict of interest.
